# Yiyi Fuzi Baijiang Powder Alleviates Dextran Sulfate Sodium-Induced Ulcerative Colitis in Rats via Inhibiting the TLR4/NF-*κ*B/NLRP3 Inflammasome Signaling Pathway to Repair the Intestinal Epithelial Barrier, and Modulating Intestinal Microbiota

**DOI:** 10.1155/2023/3071610

**Published:** 2023-01-14

**Authors:** Jinguang Yang, Lili Miao, Ye Xue, Xiaoyan Wang

**Affiliations:** ^1^The First Clinical Medical College, Shandong University of Traditional Chinese Medicine, Jinan, Shandong, China; ^2^Experimental Center of Shandong University of Traditional Chinese Medicine, Jinan, Shandong, China; ^3^College of Traditional Chinese Medicine, Shandong University of Traditional Chinese Medicine, Jinan, Shandong, China

## Abstract

Ulcerative colitis (UC) is a chronic non-specific inflammatory disease of the intestine, which is prone to recurrence and difficult to cure. Yiyi Fuzi Baijiang powder (YFBP), as a classic Chinese herbal formula, is commonly used in the clinical treatment of UC. However, its potential mechanism remains unclear. In this study, we investigated the mechanism by which YFBP exerts a therapeutic effect against UC. Firstly, we used network pharmacology to screen the active ingredients and potential targets of YFBP and constructed a “drug-ingredient-target” network. Based on bioinformatics, we searched for differentially expressed genes (DEGs) associated with UC and obtained common targets. The core targets of YFBP in the treatment of UC were identified using a protein-protein interaction (PPI) network, and molecular docking techniques were used to evaluate the binding energies of the core targets and corresponding ingredients. Enrichment analysis by Gene Ontology (GO) and Kyoto Encyclopedia of Genes and Genomes (KEGG) revealed that YFBP exerted therapeutic effects by regulating multiple inflammatory pathways including TLR4, NF-*κ*B, and TNF. Secondly, an experimental study was carried out in vivo for verification. Our results demonstrated that YFBP could effectively improve the symptoms and intestinal pathological of UC rats. Further study showed that YFBP could significantly downregulate the expressions of TLR4 and p-NF-*κ*B p65 in UC rats, inhibit the activation of NLRP3 inflammasome, reduce the levels of IL-1*β* and TNF-*α*, and then upregulate the expressions of tight junction proteins in intestinal epithelial cells. In addition, YFBP could improve the intestinal microbial community. In conclusion, our study revealed that YFBP had a good therapeutic effect on UC, and its mechanism might be related to the inhibition of the TLR4/NF-*κ*B/NLRP3 inflammasome signaling pathway to repair intestinal epithelial barrier and the modulation of intestinal microbiota.

## 1. Introduction

Ulcerative colitis (UC) is a chronic non-specific inflammatory disease of the intestine, mainly involving the mucosa and submucosa of the large intestine [1]. Its clinical symptoms are recurrent diarrhea, abdominal pain, mucopurulent, and bloody stool, and systemic poisoning symptoms may occur in severe cases [2]. In addition, UC may also affect multiple extraintestinal organs and systems, accompanied by enteropathic arthropathy, skin and mucous membrane, and eye diseases, which seriously affect the daily life and work of patients [3]. Although its specific pathogenesis is still unclear, more and more studies have shown that the pathogenesis of UC is mainly related to immune dysfunction, impaired intestinal epithelial barrier, changes in intestinal microbiota, and individual genetic susceptibility [4]. Currently, glucocorticoids, aminosalicylic acid preparations, and immunosuppressants are mainly used in clinical treatments for UC, but these drugs have serious side effects and are difficult to control its recurrence. The incidence of UC has been increasing worldwide in recent years, especially in developing and newly industrialized countries [5]. Therefore, it is urgent to find new ways to treat UC.

Traditional Chinese medicine (TCM) has obvious advantages in treating UC by the combination of syndrome differentiation and disease differentiation. Yiyi Fuzi Baijiang powder (YFBP), a classic Chinese herbal formula in the Synopsis of the Golden Chamber written by Zhang Zhongjing of the Han Dynasty, is composed of Coix lacryma-jobi var. ma-yuen (Rom. Caill.) Stapf (CS, Yiyiren), *Aconitum carmichaelii* Debeaux (AD, Fuzi), and *Patrinia villosa* (Thunb.) Juss (PJ, Baijiangcao) (Table 1). It has the effects of resolving dampness, relieving diarrhea, expelling pus, and removing toxin. In recent years, YFBP has been found to have a variety of pharmacological activities, including anti-inflammatory, anticancer, and improving immunity [6–8]. Previously, we applied YFBP to the clinical treatment of UC, which achieved good clinical efficacy and reduced the recurrence rate [9, 10]. However, its specific mechanism has not yet been fully elucidated.

Based on the theory of system biology, network pharmacology constructs a network of “disease-gene-target-drug” interaction and explains the complex mechanism between drugs and the human body. The principle of network pharmacology is in line with the concept of wholism of TCM, which is helpful to explore the complex mechanism of Chinese herbal formula [11]. Bioinformatics, based on genomics, can accurately and efficiently screen the pathogenic genes of diseases by analyzing of a large amount of genetic data, which plays an important role in deeply excavating the potential mechanisms and core targets of diseases [12]. The combined application of network pharmacology and bioinformatics can provide data support for experimental research, help to discover the potential bioactive ingredients and action targets of traditional Chinese herbal formula more accurately and efficiently, and help to comprehensively explain the relevant mechanisms of drug treatments.

Therefore, we combined network pharmacology and bioinformatics to explore the potential pathways and targets of YFBP in the treatment of UC and verified them through the DSS-induced UC rat model, providing a beneficial reference for further elucidating the mechanism of YFBP.

## 2. Materials and Methods

### 2.1. Drugs and Reagents

DSS was the product of MP Biomedicals (molecular weight: 36000-50000, Aurora, Ohio, USA). *Aconitum carmichaelii* Debeaux and Coix lacryma-jobi var. ma-yuen (Rom.Caill.) Stapf were obtained from Huqiao Pharmaceutical Co., Ltd., China. *Patrinia villosa* (Thunb.) Juss was obtained from Shandong Baiweitang Pharmaceutical Co., Ltd., China. Anti-TLR4 antibody (sc-293072), anti-ASC antibody (sc-514414), anti-ZO-1 antibody (sc33725), and anti-claudin 1 antibody (sc-166338) were purchased from Santa Cruz Biotechnology, Inc. (Texas, USA). Anti-phos-NF-*κ*B p65 antibody (3033), anti-NF-*κ*B p65 (8242), anti-occludin antibody (91131), anti-GSDMD antibody (39754), and anti-caspase-1 antibody (83383) were purchased from Cell Signaling Technology, Inc. (Boston, USA). ECL (A38555), DAPI (62248), and anti-NLRP3 antibody (PA5-79740) were purchased from Thermo Fisher Technology Co., Ltd. (Pittsburgh, PA, USA). Anti-GSDMD antibody (NBP2-80427) was purchased from Novus Biologicals, Inc. (Colorado, USA). Secondary antibodies and anti-GAPDH antibody (ab8245) were purchased from Abcam Company (Cambridge, UK). Rat IL-1 *β*/IL-1F2 Quantikine ELISA Kit (RLB00) and rat TNF-*α* ELISA Kit (RTA00) were obtained from R&D Systems (Minnesota, USA).

### 2.2. Target Prediction of YFBP Active Ingredients and UC

The traditional Chinese medicine system pharmacology database TCMSP (https://tcmspw. com/tcmsp. php) was used to screen drugs with oral bioavailability (OB) ≥ 30% and drug likeness (DL) ≥ 0.18, to obtain the active ingredients of CS, AD, and PJ and sorted out their corresponding targets. The obtained target names were converted into gene names using the UniProt database (https://sparql.uniprot.org/). Above results were sorted and imported into Cytoscape 3.8.2 software to construct the “drug-ingredient-target” network of YFBP and analyzed and sorted them.

The differentially expressed genes (DEGs) of UC were screened by using the GEO database (https://www.ncbi.nlm.nih.gov/geo/) and the “limma” package in R language (R 4.1.3), and the screening criteria were |Log *FC*| > 2 and *p* value (*Q* value) < 0.05. Then, the volcano plot and heatmap were drawn. The Venn map (https://bioinformatics.psb.ugent.be/webtools/Venn/) was constructed to obtain co-targets between YFBP and DEGs and imported co-targets into the STRING database (https://cn.string-db.org/) to build a PPI network. The PPI network was further optimized by Cytoscape software 3.8.2, the co-targets were sorted by R language according to the degree value, and the core targets were filtered. The Gene Ontology (GO) and Kyoto Encyclopedia of Genes and Genomes (KEGG) enrichment analysis of the co-targets were enriched by DAVID database (http://david.abcc.ncifcrf.gov/home.jsp, version 6.8). During the analysis, the species was selected as “Homo sapiens”, and *p* < 0.05 was set. Finally, the bioinformatics database (http://www.bioinformatics.com.cn/) was used to visualize the results.

### 2.3. Molecular Docking

The Mol2 format files of YFBP active ingredients were downloaded from the TCMSP database. The PDB format file of core targets were downloaded from the PDB database (https://www.rcsb.org), and the water molecules and protein residues were removed from their protein structures by PyMOL software. Organic compounds and core targets were processed using AutoDock Tools software and converted to PDBQT format. Molecular docking was done by using AutoDock Vina 1.5.6 software, and the results were further analyzed according to binding energy. The interaction relationship between YFBP active ingredients and core targets was visualized using PyMOL software, and the 2D structural map was derived.

### 2.4. Animals and Experimental Design

Twenty-four male Sprague-Dawley (SD) rats (6 weeks old, 160-180 g) were supplied by the Beijing Vital River Laboratory Animal Technology Co., Ltd., China (SCXK (Jing) 2021-0011). All animals were kept in a controlled environment (room temperature: 20 ± 2°C, relative humidity: 50%-70%, and a 12 h light-dark cycle) and were fed with a standard diet. After one week of adaptive feeding, the rats were randomly divided into three groups (*n* = 8): the normal group, the DSS group, and the DSS+YFBP group. The rats of the normal group were allowed to drink purified water freely. The rats in the DSS group and the DSS+YFBP group freely drank fresh 5% DSS solution for 7 days and then changed to 3% DSS solution. After the DSS-induced acute UC models were successfully established, the drinking water of the DSS group and the DSS+YFBP group was changed to purified water. The rats in the DSS+YFBP group were given YFBP 4.59 g/kg/d by gavage for 7 days, while the rats in the normal group and the DSS group were given normal saline 4 ml by gavage every day. After the final gavage, the rats were anesthetized with isoflurane gas, and blood was collected from the abdominal aorta and centrifuged at 3000 rpm at 4°C for 15 min. We took the supernatant and stored it at -80°C. The colon tissue and feces were taken out and stored at -80°C for further detection. All animal experiment protocols were approved by the Ethics Committee of Shandong University of Traditional Chinese Medicine (Jinan, China) (approval number: SDUTCM20211022001).

### 2.5. Assessment of the Disease Activity Index (DAI)

During the experiment, the body weight, fecal characteristics, and blood in the stool of rats were recorded every day, and each index was scored. The specific scores are as follows: percentage of weight loss (0: no loss; 1: 1-5%; 2: 6-10%; 3: 11-15%; and 4: more than 15%), fecal character (0: normal; 1: wet feces; 2: pasty feces; 3: semi liquid; and 4: watery feces), and stool blood (0: normal; 1: slight bleeding; 2: moderate bleeding; 3: severe bleeding; and 4: gross bleeding). These scores were added and then divided by 3 to obtain the DAI. Finally, the severity of UC was evaluated by the DAI score [13].

### 2.6. Histological Analysis of Colon

The colonic tissues of rats were fixed with 4% paraformaldehyde for 48 h and then embedded in paraffin and sliced (thickness 5 *μ*m). The sections were dewaxed and washed after baking at 60°C for 3 hours and stained with hematoxylin and eosin (H&E). Finally, they were observed and photographed with a phase contrast microscope (Olympus, Japan).

### 2.7. UPLC-Q-Orbitrap HRMS/MS Analysis

The analysis was performed using UPLC-Q-Orbitrap HRMS/MS (UltiMate 3000 RS) chromatograph and Q Exactive high-resolution mass spectrometer (Thermo Fisher Technology Co., Ltd., China). Column: AQ-C18 (150 × 2.1 mm, 1.8 *μ*m), aqueous phase: 0.1% formic acid aqueous solution, and organic phase: methanol. Gradient elution: 0-1 min, 2% B; 1-5 min, 20% B; 5-10 min, 50% B; 10-15 min, 80% B; 15-25 min, 95% B; and 25-30 min, 2% B; the flow rate during elution was 0.3 ml/min, the injection volume was 5.0 *μ*l, and the MS acquisition was performed by the scanning mode of positive and negative ion switching. The specific conditions of mass spectrometry: full mass/dd-MS2 detection mode, electrospray ionization source (ESI) voltage of +3.8 kV, capillary temperature of 300°C, sheath gas flow of 45 Arb, and auxiliary gas flow of 15 Arb. The scanning range was 150.0-2000.0 m/z, the primary resolution was 70 000 (full mass), the secondary resolution was 17 500 (dd-MS2), and the ion source temperature was 350°C [14].

### 2.8. Enzyme-Linked Immunosorbent Assay (ELISA)

The colon tissue was weighed and cut into pieces and then put into a homogenizer. Precooled PBS was added into the homogenizer at a ratio of 1 : 10 (g/ml) for homogenization; then, a homogenizer was placed on ice, and the homogenization was repeated several minutes later. After the homogenate was placed on ice and cracked for 30 min, it was centrifuged at 12000 rpm at 4°C for 5 min, and the supernatant was taken. The levels of inflammatory cytokines, including IL-1*β* and TNF-*α*, in the supernatant were assessed by corresponding ELISA kits. The experiments were performed according to the manufacturer's instructions, and the OD values were measured at 450 nm using a microplate reader.

### 2.9. Western Blotting Assay

Colon tissue was lysed with RIPA lysate, and total protein was extracted. The protein concentration was determined with a BCA protein detection kit. Proteins were separated by 12% sodium dodecyl sulfate-polyacrylamide gel and transferred to the PVDF membrane. After washing twice, the membranes were blocked with Tris-buffered saline Tween 20 (TBST) containing 5% skimmed milk powder at 25°C for 1 h and then incubated overnight at 4°C with primary antibodies: TLR4, phos-NF-*κ*B p65, NF-*κ*B p65, NLRP3, ASC, caspase-1, GSDMD, ZO-1, claudin-1, occludin (all 1 : 1000), and GAPDH (1 : 10000). The membranes were washed three times with TBST and incubated with secondary antibodies at 25°C for 1 h and then washed three times with TBST. After the membrane protein was fully contacted with chemiluminescence reagent for 5 min, it was detected by Tanon 6600 luminescence imaging workstation, and the optical density value was analyzed by Image-Pro Plus 6.0 software.

### 2.10. Immunofluorescence Assay

The dewaxed colon tissue sections were placed in citrate buffer for antigen retrieval in a high-temperature environment. After washing 3 times with PBS, the sections were blocked and incubated in 5% BSA for 30 min and then cultured overnight at 4°C with primary antibodies: NLRP3 (1 : 250), ZO-1 (1 : 500), and GSDMD-N (1 : 50). After being washed with PBS for 3 times, the sections were incubated with secondary antibody (1 : 1000) in a dark environment at room temperature for 60 min. Finally, the sections were incubated with 4′,6-diamino-2-phenylindole (DAPI) solution in a dark environment for 5 min, resin sealed and detected by a laser scanning confocal microscope (LSM; Zeiss, Germany), and the optical density values were analyzed by Image-Pro Plus 6.0 software.

### 2.11. 16S rDNA Sequencing and Intestinal Microbiota Analysis

Microbial DNA was extracted using the HiPure Stool DNA Kits (Magen, Guangzhou, China) according to manufacturer's protocols. The 16S rDNA V3-V4 region of the ribosomal RNA gene was amplified by PCR (94°C for 2 min, followed by 30 cycles at 98°C for 10 s, 62°C for 30 s, and 68°C for 30 s, and a final extension at 68°C for 5 min) using primers 341F: CCTACGGGNGGCWGCAG and 806R: GGACTACHVGGGTATCTAAT. Amplicons were extracted from 2% agarose gels, purified using the AxyPrep DNA Gel Extraction Kit (Axygen Biosciences, Union City, CA, USA) according to the manufacturer's instructions, and then quantified using ABI StepOnePlus Real-Time PCR System (Life Technologies, Foster City, USA). Purified amplicons were pooled in equimolar and paired-end sequenced (2 × 250) on an Illumina platform according to the standard protocols. Biomarker features in each group were screened by LEfSe software (version 1.0) and randomRorest package (version 4.6.12) in R language.

### 2.12. Statistical Analysis

All data were shown as mean ± standard deviation (SD) in the experiments. Statistical analysis of intestinal microbiota including Welch's *t*-test, Wilcoxon rank test, Tukey's HSD test, Kruskal-Wallis H test, and Adonis and Anosim tests was performed using R language vegan package (version 2.5.3). SPSS 22.0 software was used for one-way analysis of variance (ANOVA) followed by the Fisher's least significant difference test for multiple comparisons. Unpaired *T*-test was also used to determine significant differences between two groups of data. *p* <0.05 was considered to be statistically significant between groups.

## 3. Results

### 3.1. Targets Corresponding to Active Ingredients of YFBP and DEGs of UC

**Figure 1:**
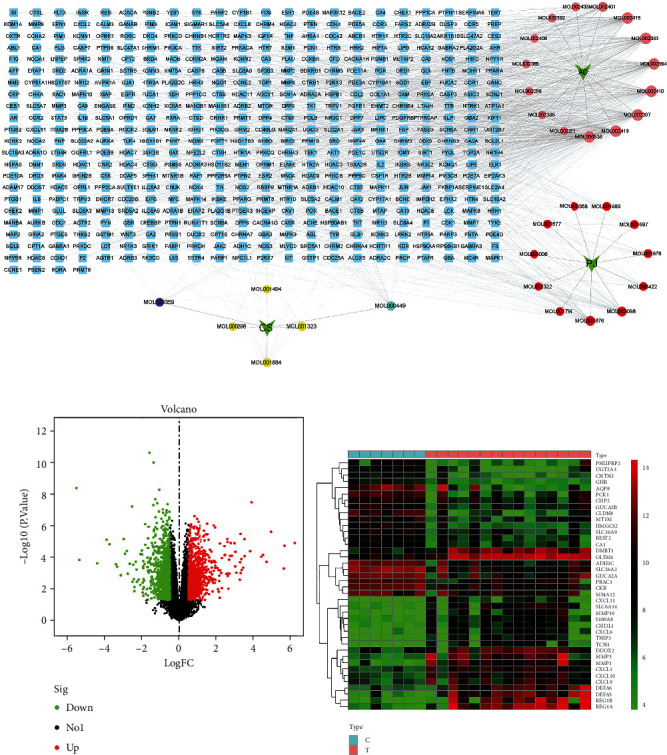
Drug-ingredient-target network and DEGs related to UC in the GEO database. (a) Yellow nodes were CS ingredients, pink nodes were AD ingredients, and red nodes were PJ ingredients. MOL000359 was in CS, AD, and PJ. MOL000449 was in CS and PJ. The larger the node, the greater the degree value, indicating that the interaction between the target and the ingredient was stronger. (b) Volcano plot of DEGs, where red dots indicated upregulated genes, and green dots indicated downregulated genes. (c) Heatmap. Red dots were upregulated expression, and green dots were downregulated expression.

Using the TCMSP database, we found a total of 40 active ingredients in the YFBP formula (Supplementary Table 1), and the UniProt database was used to standardize and organize the corresponding targets of the active ingredients. Finally, 487 effective targets were identified. The above results were imported into Cytoscape 3.8.2, and a network graph of “drug-ingredient-target” consisting of 523 nodes and 1035 edges was constructed (Figure 1(a)).

**Figure 2:**
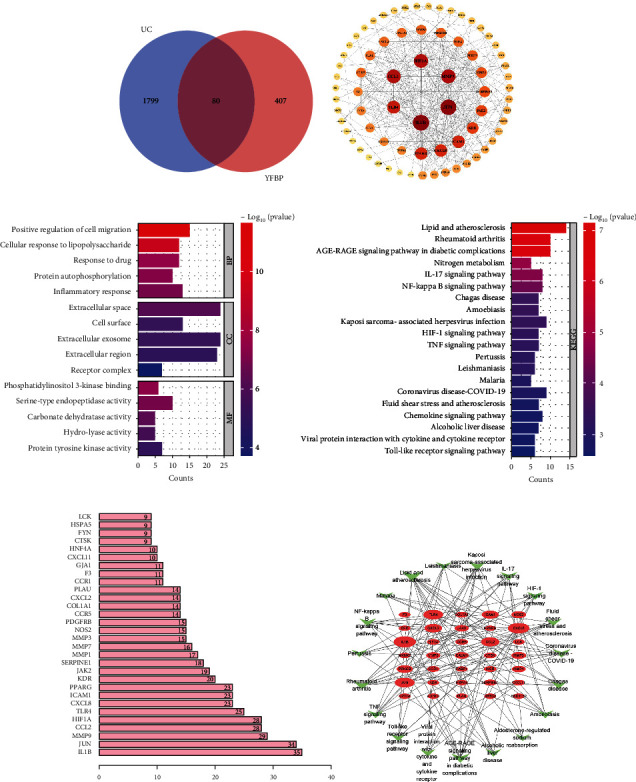
Co-targets prediction and analysis. (a) The Venn diagram of common targets between YFBP and UC. (b) PPI network of common targets. (c) GO analysis and KEGG analysis of the co-targets of YFBP in treatment of UC using a bar graph to rank the importance from top to bottom. (d) The number of adjacent nodes of the top 30 co-targets of YFBP in treatment of UC. (e) Target-pathway network. The co-targets of YFBP in treatment of UC were shown as a pink oval node. The higher the degree of the target, the larger the size. Signaling pathways were marked with green triangle nodes.

Relying on the R language, we screened 1879 UC DEGs from the GSE36807 dataset, of which 984 were upregulated and 895 were downregulated. The volcano plot of all DEGs was drawn (Figure 1(b)), and the 20 genes with the most significant upregulation and downregulation were selected, respectively, to draw heatmap (Figure 1(c)). Eighty co-targets were found between YFBP and UC by Venn diagram (Figure 2(a)). The PPI network of the co-targets is shown in Figure 2(b). The darker the color, the larger the node area, indicating that the higher the degree of association and importance of the gene. The top 30 targets in co-targets with the highest correlation are showed in Figure 2(d). According to the above analysis, we found that the core targets of UC were IL-1*β*, JUN, MMP9, HIF1*α*, CCL2, and TLR4.

GO and KEGG analyses were performed by the R language (Figure 2(c)). GO enrichment analysis showed that YFBP mainly acted on the extracellular space, extracellular exosome, and extracellular region and affected the cellular response to lipopolysaccharide, positive regulation of cell migration, and other biological processes by affecting the phosphatidylinositol 3-kinase binding and serine-type endopeptidase activity. The results of KEGG analysis indicated that the NF-*κ*B signaling pathway, IL-17 signaling pathway, and TLR signaling pathway were closely related to core targets. Finally, by constructing a target pathway network (Figure 2(e)), we found that YFBP could regulate disease targets through multiple inflammatory and immune pathways, thereby exerting effects on UC.

### 3.2. The Results of Molecular Docking

**Figure 3:**
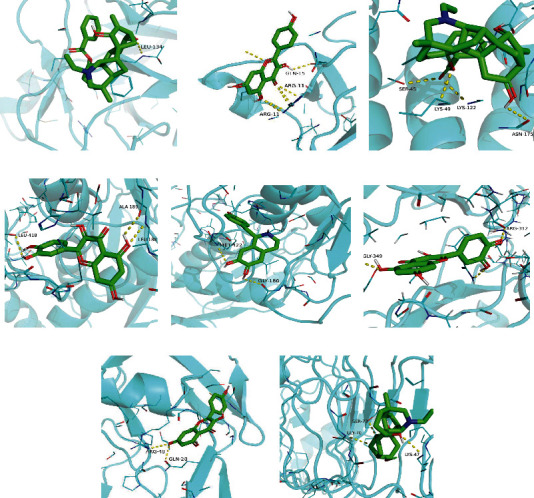
Partial diagram of molecular docking. (a) IL-1*β*-benzoylnapelline. (b) IL-1*β*-kaempferol. (c) JUN-benzoylnapelline. (d) MMP9-quercetin. (e) MMP9-(R)-norcoclaurine. (f) HIF1*α*-quercetin. (g) CCL2-kaempferol. (h) TLR4-karakoline.

According to the above studies, we found that the main active ingredients of YFBP were MOL000538, MOL002419, MOL002397, MOL002410, MOL002394, MOL001676, MOL000098, and MOL000422 (Figure 1(a)), and the core targets of UC were IL-1*β*, JUN, MMP9, HIF1*α*, CCL2, and TLR4. The proteins 6y8i (IL-1*β*), 6y3v (JUN), 1gkc (MMP9), 5las (HIF1*α*), 7so0 (CCL2), and 2z62 (TLR4) corresponding to the above core targets were downloaded from the RCSB PDB database as receptors. Molecular docking of receptor protein and active ingredient ligand was carried out with AutoDock Vina software. Five effective active ingredients were successfully verified and docked. By further calculating the binding energy, we found that the binding energies of all ligands and receptors were <-5.0 kcal/mol (Supplementary Table 2). It is generally believed that the binding energy <0 kcal/mol indicates that the ligand and receptor can bind naturally. The smaller the binding energy is, the more stable the structure between them is. The results of molecular docking also showed that the core targets had good binding ability to the main ingredients of YFBP such as quercetin, benzoylnapelline, kaempferol, (R)-norcoclaurine, and karakoline. Finally, we visualized the structural diagrams of molecular docking through pyMOL (Figure 3).

### 3.3. Effects of YFBP on Disease Status and Colon Pathology in UC Rats

**Figure 4:**
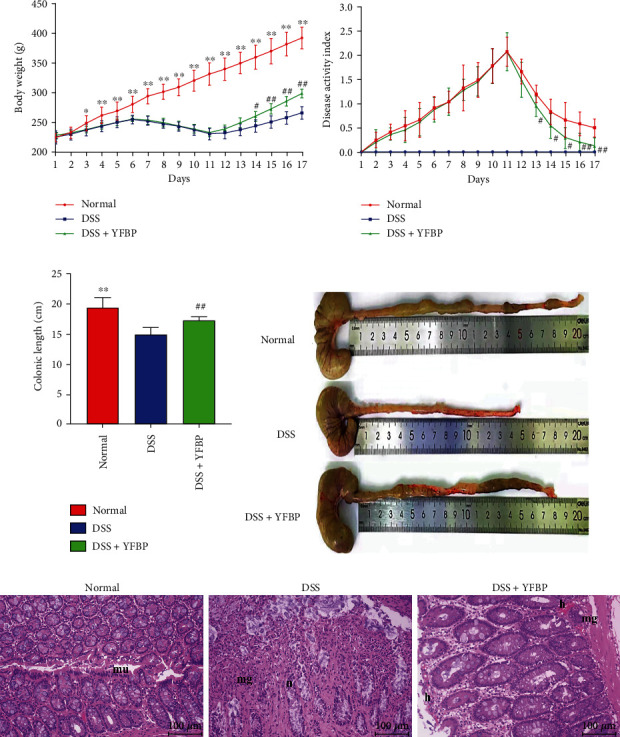
Effects of YFBP on disease status and colon pathology of UC rats. Changes of body weight (a) and DAI score (b) of rats in each group during the experiment (*n* = 8). (c) The lengths of colons of each group of rats (*n* = 8). (d) Representative images of colons from each group. (e) Representative images of H&E staining of colonic mucosa in each group (mu: mucosal layer; mg: focal aggregation of inflammatory cells; h: hemorrhage; n: necrosis; *n* = 3); magnification = 200x, scale bar 100 *μ*m. All values were presented as mean ± SD. ^∗^*p* < 0.05, ^∗∗^*p* < 0.01, normal vs. DSS group; ^#^*p* < 0.05, ^##^*p* < 0.01, DSS+YFBP vs. DSS group.

The body weight and DAI score of rats in each group were measured every day. As time went on, the body weight gain of DSS-treated rats decreased, and the weight loss started on the seventh day. After the intervention of YFBP, the body weight gradually increased. From the 14th day of the experiment, there was a statistical difference between the DSS+YFBP group and the DSS group (Figure 4(a) and Supplementary Table 3). The DAI score of the DSS-treated rats increased day by day, reaching the peak on the 11th day, and all rats developed diarrhea and bloody stool. After YFBP treatment, the DAI score gradually decreased. From the 13th day of the experiment, there was a statistical difference compared with the DSS group (Figure 4(b) and Supplementary Table 4). Subsequently, we measured the colon length of the rats and found that the colon length of the DSS group was significantly shortened, and there was a statistical difference compared with the normal group (*p* < 0.01). YFBP intervention could reverse this change to some extent (*p* < 0.01) (Figures 4(c) and 4(d) and Supplementary Table 5). Histological analysis showed that the colonic mucosal epithelium of rats in the DSS group was absent and irregularly arranged, with a large number of inflammatory cells, lymphocytes aggregated, goblet cells decreased, and accompanied by necrosis. However, in the DSS+YFBP group, the epithelium around the colon mucosa was proliferated and repaired, the glands were arranged relatively regularly, the inflammatory cell infiltration and lymphocyte aggregation were reduced, the goblet cells were abundant, and the bleeding points were reduced (Figure 4(e)). These indicated that YFBP could significantly alleviate the disease status and colon injury in UC rats.

### 3.4. Chemical Constituents of YFBP-Containing Serum

**Figure 5:**
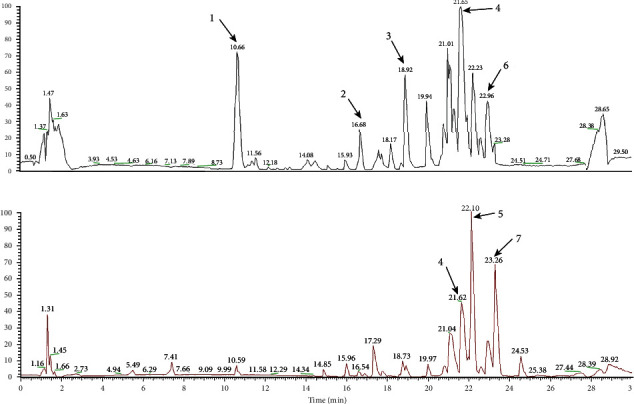
Total ion chromatogram of YFBP-containing serum in positive (a) and negative (b) modes. Peak 1: methyl 1-(hexopyranosyloxy)-5-hydroxy-7-(hydroxymethyl)-1,4a,5,7a-tetrahydrocyclopenta[c]pyran-4-carboxylate; 2: 6-ketoprostaglandin F1*α*; 3: caffeic acid; 4: pinolenic acid; 5: *γ*-linolenic acid ethyl ester; 6: palmitic acid; 7: *trans*-petroselinic acid.

In order to further clarify the main ingredients of YFBP that play a therapeutic role, the chemical constituents of YFBP-containing serum were analyzed by the UPLC-Q-Orbitrap HRMS method. The results are shown in Figure 5 and Supplementary Table 6. A total of seven chemical constituents were identified using information on fragmentation, literature, and excimer peaks, including methyl 1-(hexopyranosyloxy)-5-hydroxy-7-(hydroxymethyl)-1,4a,5,7a-tetrahydrocyclopenta[c]pyran-4-carboxylate, 6-ketoprostaglandin F1*α*, caffeic acid, pinolenic acid, *γ*-linolenic acid ethyl ester, palmitic acid, and *trans*-petroselinic acid.

### 3.5. YFBP Inhibited TLR4/NF-*κ*B/NLRP3 Inflammasome Signaling Pathway

**Figure 6:**
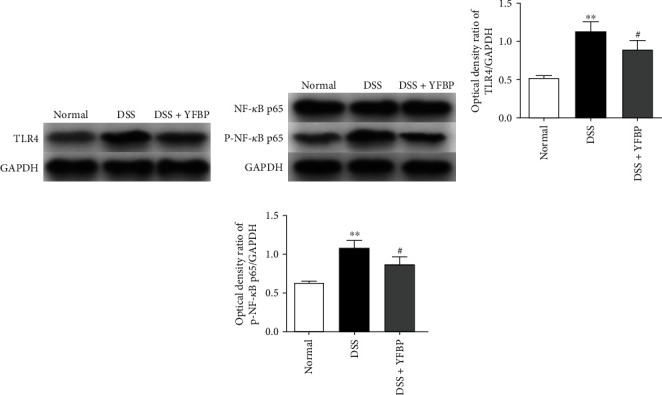
Effects of YFBP on the expressions of TLR4 and NF-*κ*B in UC rats. (a, b) The protein expressions of TLR4 and p-NF-*κ*B p65 in colon tissue of UC rats were detected by western blotting assay, and representative bands were shown. The relative expressions of TLR4 (c) and p-NF-*κ*B p65 (d), respectively (*n* = 3). All values were presented as mean ± SD. ^∗∗^*p* < 0.01 vs. the normal group; ^#^*p* < 0.05 vs. the DSS group.

**Figure 7:**
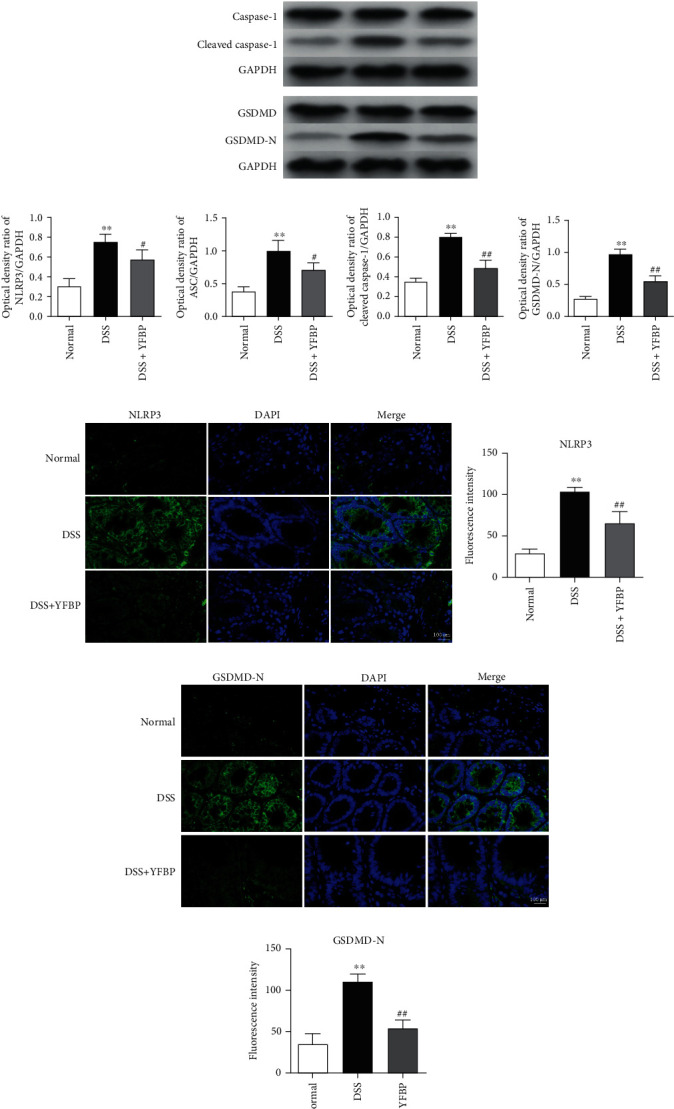
Effects of YFBP on the NLRP3 inflammasome in the colon. (a) The protein expressions of NLRP3, ASC, cleaved caspase-1, and GSDMD-N in the colon tissue of UC rats were detected by western blotting assay, and representative bands were shown. The relative expressions of NLRP3 (b), ASC (c), cleaved caspase-1 (d), and GSDMD-N (e), respectively (*n* = 3). Immunofluorescence staining of NLRP3 (f) and GSDMD-N (h) in the colon. Nuclei were stained with DAPI, magnification = 630x, scale bar 100 *μ*m. The images showed quantification of NLRP3 (g) and GSDMD-N (i) expressions (*n* = 3). All values were presented as mean ± SD. ^∗∗^*p* < 0.01 vs. the normal group; ^#^*p* < 0.05 and ^##^*p* < 0.01 vs. the DSS group.

The activation of the TLR4/NF-*κ*B/NLRP3 inflammasome signaling pathway is closely related to the pathogenesis of UC. Here, we quantified the expressions of key proteins of the TLR4/NF-*κ*B/NLRP3 inflammasome signaling pathway using western blotting. As shown in Figure 6 and Supplementary Table 7, the protein expressions of TLR4 and p-NF-*κ*B p65 in the colon of DSS-induced UC rats were significantly increased (both *p* < 0.01) when compared to the normal group. After administration, the expressions of TLR4 and p-NF-*κ*B p65 were significantly decreased in the DSS+YFBP group (both *p* < 0.05). The expression levels of NLRP3, ASC, cleaved caspase-1, and GSDMD-N in the colon of UC rats were significantly higher than those in the normal group (all *p* < 0.01). After YFBP treatment, the expression levels of NLRP3, ASC, cleaved caspase-1, and GSDMD-N were significantly decreased when compared to the DSS group (Figures 7(a)–7(e) and Supplementary Table 7). In addition, we analyzed the expressions of NLRP3 and GSDMD-N by immunofluorescence analysis, and similar results were obtained (Figures 7(f)–7(i) and Supplementary Table 8). These results indicate that the therapeutic effect of YFBP on UC may be achieved by inhibiting the activation of TLR4/NF-*κ*B/NLRP3 inflammasome signaling pathway.

### 3.6. YFBP Reduced the Levels of Proinflammatory Cytokines

**Figure 8:**
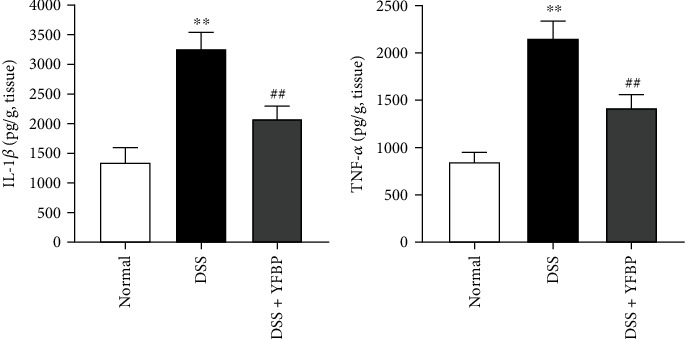
Detecting the levels of inflammatory cytokines in colon by ELISA (*n* = 3). All values were presented as mean ± SD. ^∗∗^*p* < 0.01 vs. the normal group; ^##^*p* < 0.01 vs. the DSS group.

The levels of proinflammatory cytokines in the colon tissue of rats were detected by ELISA. The results are shown in Figure 8 and Supplementary Table 9, compared with the normal group, the expression levels of cytokines such as IL-1*β* and TNF-*α* in the colon tissue of the DSS-induced UC rats significantly increased (both *p* < 0.01). However, the expression levels of IL-1*β* and TNF-*α* decreased significantly in the DSS+YFBP group after treatment (both *p* < 0.01). These results showed that YFBP was able to regulate the overexpression of inflammatory cytokines in UC rats.

### 3.7. YFBP Increased the Expressions of Tight Junction (TJ) Proteins in the Intestinal Epithelium

**Figure 9:**
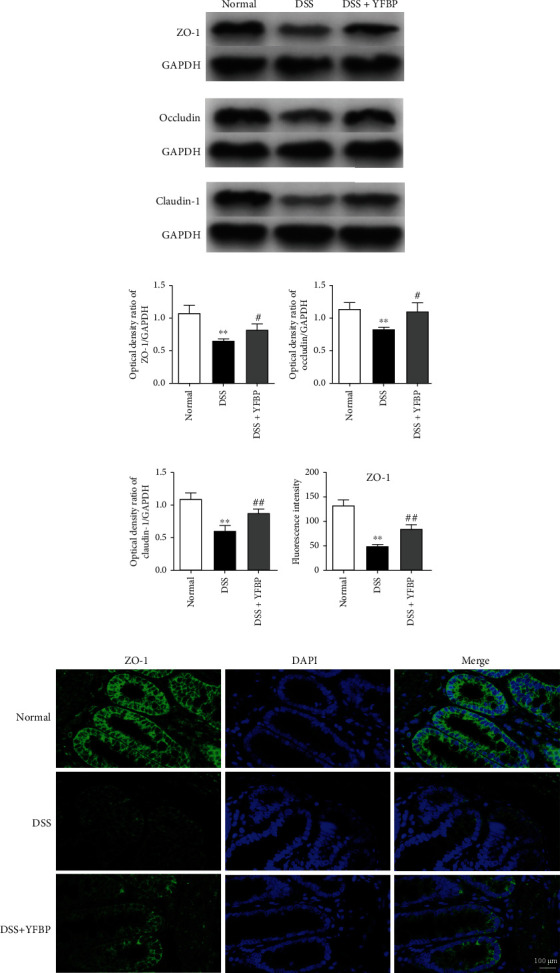
Effects of YFBP on the TJ of intestinal epithelium. (a) The protein expressions of ZO-1, occludin, and claudin-1 in colon were detected by western blotting assay, and representative bands were shown. The relative expressions of ZO-1 (b), occludin (c), and claudin-1 (d), respectively (*n* = 3). (f) Immunofluorescence staining of ZO-1 in colon. Nuclei were stained with DAPI, magnification = 630x, and scale bar 100 *μ*m. (e) The image showed quantification of ZO-1 expression (*n* = 3). The results were presented as mean ± SD. ^∗∗^*p* < 0.01 vs. the normal group; ^#^*p* < 0.05 and ^##^*p* < 0.01 vs. the DSS group.

To further investigate whether YFBP has a protective effect on the intestinal epithelium of UC rats, we detected the protein expressions of ZO-1, occludin, and claudin-1 by western blotting and immunofluorescence. Compared with the normal group, the protein expressions of ZO-1, occludin, and claudin-1 in colon of UC rats significantly decreased (all *p* < 0.01). However, YFBP remarkably enhanced the expressions of ZO-1 (*p* < 0.05), occludin (*p* < 0.05), and claudin-1 (*p* < 0.01) (Figures 9(a)–9(d) and Supplementary Table 7). Consistently, as shown in Figures 9(e) and 9(f), decreased flourescence intensity in the DSS group was observed, indicating lower expression of ZO-1 (*p* < 0.01), and YFBP treatment reversed the abundance of this protein (*p* < 0.01) (Supplementary Table 8). These results suggested that YFBP might repair intestinal injury by regulating the TJ of intestinal epithelium.

### 3.8. YFBP Regulated the Intestinal Microbiota

**Figure 10:**
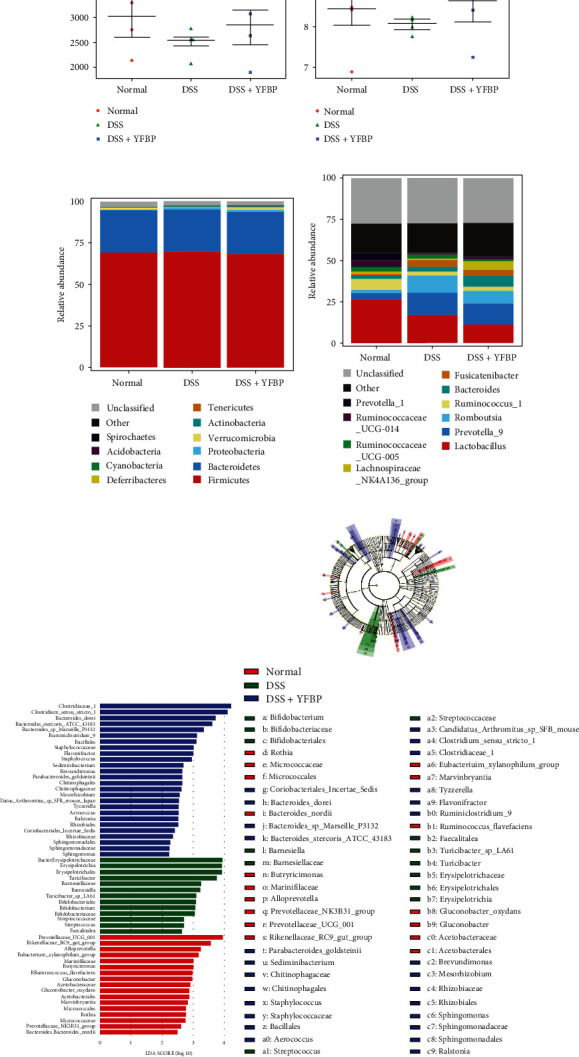
Effects of YFBP on the intestinal microbiota of UC rats. The Chao1 indexes (a) and Shannon indexes (b). Bar graphs of intestinal microbiota at the phylum (c) and genus (d) levels. (e) Differentially enriched intestinal microbiota in all groups by linear discriminant analysis (LDA). LDA score higher than 3.0 indicated that the abundance of this group was higher than that of other groups. (f) Cladogram based on LEfSe analysis.

We detected the intestinal microbiota of rats by 16S rDNA high-throughput sequencing. The Chao 1 and Shannon indexes indicated that the diversity and richness of intestinal microbiota in DSS-induced UC rats were significantly lower than those in the normal group (Figures 10(a) and 10(b)). Compared with the DSS group, the diversity and richness of intestinal microbiota increased after YFBP treatment.

The histogram analysis of the intestinal microbial community showed that *Firmicutes* and *Bacteroidetes* were dominant groups at the phylum level in all experimental groups (Figure 10(c)). Compared with the normal group, the proportion of Proteobacteria in the DSS group increased, and the proportion of *Verrucomicrobia* decreased. However, the above changes could be reversed to a certain extent after YFBP treatment. At the genus level (Figure 10(d)), compared with the normal group, the proportion of Prevotella_9 in the DSS group increased, and the proportion of the Lachnospiraceae_NK4A136_group genera decreased. In the DSS+YFBP group, the proportion of Prevotella_9 decreased and the proportion of Lachnospiraceae_NK4A136_group increased after treatment. The above results showed that YFBP could increase the beneficial bacteria in the intestinal tract, reduce the pathogenic bacteria, and maintain the balance of intestinal microbiota in UC rats.

To identify bacterial taxa with significant differences in different experimental groups, linear discriminant analysis effect size (LEfSe) was used to detect the intestinal microbiota of rats. As shown in Figure 10(e), a total of 56 species with significant differences were obtained from the three groups, among which the most abundant differential taxa in the normal group were Prevotellaceae_UCG_001 and Rikenellaceae_RC9_gut_group. *Erysipelotrichaceae*, *Turicibacter*, and Barnesiellaceae were the most abundant differential taxa in the DSS group. Meanwhile, Clostridiaceae_1 and Bacteroides_dorei were enriched in the DSS+YFBP group. The cladogram further revealed key bacterial changes in each experimental group (Figure 10(f)). These results suggested that YFBP treatment altered the composition and population structure of intestinal microbiota in UC rats.

## 4. Discussion

As a classic Chinese herbal formula for the treatment of digestive system diseases, YFBP has been proven to play a role in the treatment of UC by alleviating inflammation and reducing the permeability of colonic mucosa [15, 16]. However, its specific mechanism remains to be further clarified. Chinese herbal formula has complex chemical compositions and action mechanisms. Therefore, it is difficult to analyze all its effective ingredients and mechanism using a single drug research mode. At present, the combination of network pharmacology and bioinformatics provides a new strategy for studying the pharmacological mechanism of Chinese herbal formula in the treatment of diseases. In order to explore the potential mechanism of YFBP in the treatment of UC, we identified 80 common targets of YFBP and UC using network pharmacology and bioinformatics technology, constructed the PPI network, and carried out GO enrichment analysis and KEGG analysis. Furthermore, the main ingredients in YFBP and the core targets of UC were verified by molecular docking technology. The above results indicated that YFBP could treat UC by affecting a variety of inflammatory pathways including TLR4, NF-*κ*B, and TNF. Subsequently, we carried out experiments in vivo for verification.

The rat model of UC induced by DSS is similar to human UC in clinical symptoms and pathological characteristics [17], so it is a commonly used in experimental researches. In this study, we found that YFBP significantly improved the weight loss, diarrhea, bloody stool, DAI index, colon length, and intestinal pathology in DSS-induced UC rats. Then, the chemical constituents of YFBP-containing serum were analyzed by UPLC-Q-Orbitrap HRMS method. Among them, caffeic acid, pinolenic acid, and *γ*-linolenic acid ethyl ester could reduce the levels of proinflammatory factors such as IL-1*β* and IL-6, thus playing an anti-inflammatory role [18–20].

The TLR4 signaling pathway mainly mediates innate immune and inflammatory responses and is closely related to the occurrence and progression of UC [21]. As a member of the signal transduction receptor family, TLR4 can sense the stimulation of external pathogenic factors and play a role in connecting innate immunity and specific immunity. Its most important biological function is to promote the synthesis and release of inflammatory cytokines [22]. Significantly increased expression of TLR4 was previously observed in UC patients and rats [23, 24]. As an important transcription factor downstream of the TLR4 signaling receptor, NF-*κ*B can not only induce the synthesis and release of proinflammatory cytokines and aggravate the inflammatory response [25] but also destroy the tight junction of the intestine, thereby affecting the integrity and permeability of the intestinal epithelial barrier [26, 27]. Inhibiting the activation of the TLR4/NF-*κ*B signaling pathway could significantly reduce the levels of inflammatory cytokines in the serum and colon tissue of experimental animals and alleviate the inflammatory response and intestinal epithelial cell injury [28, 29]. As a protein complex composed of NLRP3, ASC, and caspase-1, NLRP3 inflammasome has the function of recognizing external stimuli and internal danger signals and initiating an inflammatory response, playing an important role in the induction of infection and autoimmune responses [30]. NLRP3 inflammasome is an important factor in controlling the integrity of intestinal epithelium and regulating intestinal homeostasis and can serve as a platform for CASP1/caspase-1 activation and the maturation of proinflammatory cytokine IL-1*β* [31]. A previous study showed that NLRP3, a key protein in the inflammasome, could be activated by the TLR4/NF-*κ*B signaling pathway, leading to the cleavage of caspase-1 [32]. The cleaved caspase-1 could decompose GSDMD into N-terminal and C-terminal structures. In the absence of C-terminal structural control, the N-terminal structure of activated GSDMD could cause holes in the cell membrane, resulting in the release of mature proinflammatory cytokines IL-1*β* and TNF-*α* into the extracellular space, thereby aggravating the inflammatory response [33]. Meanwhile, inhibiting the activation of the NLRP3 inflammasome could effectively reduce the level of the inflammatory cytokine IL-1*β*, thereby alleviating the inflammatory response and intestinal injury in UC rats [34]. In this study, we found that the expression levels of TLR4, p-NF-*κ*B P65, NLRP3, ASC, cleaved caspase-1, and GSDMD-N were significantly increased in the colon tissue of UC rats, which could be effectively reversed by YFBP treatment. Modern pharmacology studies showed that AD, as a major component of YFBP, could reduce the inflammatory response by inhibiting the activation of the TLR4/NF-*κ*B signaling pathway, and it could also slow down pyroptosis by inhibiting the NLRP3/caspase-1 pathway [35, 36]. CS extract had the inhibitory effect on the NF-*κ*B signaling pathway and was widely used in the treatment of inflammation and tumor [37]. Luteolin of PJ could effectively inhibit the expression of NLRP3 in the colon tissue of UC mice, thereby playing a role in the treatment of UC [38].

Cytokines have the functions of participating in immune response and immune regulation and play an important role in the pathogenesis of UC [39]. Among them, IL-1*β* and TNF-*α* are considered to be the key cytokines that destroy intestinal mucosa and induce inflammation [40, 41]. IL-1*β*, as the main proinflammatory cytokine, can induce the activation of other inflammatory factors, thereby coinducing the occurrence of inflammation [42]. TNF-*α* can destroy the immune system and initiate inflammation [43]. IL-1*β* and TNF-*α* could also increase intestinal permeability and disrupt the function of intestinal barrier by reducing the expression of occludin and claudin-1, thus promoting the development of intestinal inflammation [44–46]. Previous studies found that the levels of IL-1*β* and TNF-*α* were significantly increased in the colon tissue of UC patients and rats [47, 48]. In line with the above findings, here, we also observed higher levels of IL-1*β* and TNF-*α* in the colon tissue of UC rats, and all of these were significantly reduced by YFBP administration. It was found that quercetin, a natural ingredient of PJ, was closely related to the expression of IL-1*β* [49], which was also confirmed in our molecular docking, and it could inhibit the activation of the NF-*κ*B signaling pathway and reduce the level of IL-1*β*, thereby exerting an anti-inflammatory effect. Kaempferol, a flavonoid compound in PJ, has been proven to effectively reduce the level of TNF-*α*, maintain the integrity of the intestinal epithelial barrier, and exert anti-inflammatory and antioxidant effects [50]. Previous studies have shown that the active ingredients in YFBP, such as caffeic acid, pinolenic acid, and *γ*-linolenic acid ethyl ester, have anti-inflammatory effects. Caffeic acid and pinolenic acid can exert various effects such as anti-inflammatory, antitumor, and immune regulation by inhibiting the NF-*κ*B signaling pathway and reducing the levels of IL-1*β*, IL-6, and TNF-*α* [18, 19]. As a derivative of linoleic acid metabolized in the body, *γ*-linolenic acid could promote the synthesis of prostaglandin E1, inhibit the activity of HIF1*α*, and reduce the level of IL-6, thereby exerting an anti-inflammatory effect [20, 51].

The intestinal epithelial barrier, as an important line of defense against external stimuli, has the function of maintaining intestinal homeostasis, and its functional defect increases the permeability of the intestinal [52]. TJ composed of ZO-1, occludin, and claudin-1 is a key part of the intestinal epithelial barrier and plays an important role in regulating intestinal permeability and maintaining the integrity of the intestinal epithelial barrier and immune responses [53]. Previous studies have shown that the change of colonic permeability may be an important link in the pathogenesis of UC, and inflammatory cytokines are important factors affecting intestinal permeability [54]. It has been demonstrated that IL-1*β* and TNF-*α* induced increase in intestinal TJ permeability which was regulated by the activation of NF-*κ*B [55, 56]. Significant downregulation of ZO-1, occludin, and claudin-1 has been observed in UC patients and UC mice [57, 58]. In this study, we also found that the expressions of ZO-1, occludin, and claudin-1 in the colon tissue of UC rats were significantly decreased, while their expressions were significantly increased after YFBP treatment. Kaempferol in PJ could upregulate the ZO-1 protein expression to protect the intestinal epithelial barrier and reduce the inflammatory response in UC mice [59]. Caffeic acid in PJ could effectively alleviate the increase of intestinal permeability and protect intestinal integrity by increasing the gene expressions of ZO-1 and occludin [60]. Luteolin in PJ could regulate TJ via the NF-*κ*B signaling pathway, thereby repairing intestinal barrier injury [61].

The imbalance of intestinal microbiota can affect the host immune function, destroy the integrity of the intestinal epithelial barrier, and then lead to the occurrence of intestinal diseases such as UC [62, 63]. Clinical studies have showed that the abundance and diversity of intestinal microbiota in UC patients were far lower than those in healthy people. At the same time, the relevant proinflammatory cytokines produced in the patients could further disrupt the balance of intestinal microbiota and reduce the ability of intestinal epithelial barrier to intercept harmful substances, thereby aggravating the condition of UC [64]. In this experiment, based on the Chao1 and Shannon indexes, we found that the abundance and diversity of intestinal microbiota in UC rats decreased, and the intervention of YFBP reversed the above changes to a certain extent.

Evidence suggested that *Verrucomicrobia*, as the few easily detected phylum in human intestine, had the function of maintaining the integrity of intestinal epithelial barrier and secreting antimicrobial peptides [65]. Furthermore, as the only known species of *Verrucomicrobia* that exists in human intestine, *Akkermansia muciniphila*'s abundance in UC patients decreased [66]. The enrichment of Proteobacteria could destroy the microbial community structure in the intestine and then lead to the occurrence of intestinal diseases [67]. Some members of *Prevotella* could mediate proinflammatory cytokines such as IL-6 and TNF-*α*, and their large enrichment would cause intestinal microbiota disorder and increase the probability of inflammatory diseases including UC. Its proportion in the intestine of UC patients was higher than that of healthy people [68, 69]. As a member of *Firmicutes*, *Lachnospiraceae*_NK4A136_group could produce sodium butyrate, which could inhibit the NF-*κ*B p65 signaling pathway, reduce the levels of IL-1*β* and TNF-*α*, and upregulate the expressions of ZO-1 and claudin-1, thereby reducing intestinal permeability [70]. *Lachnospiraceae*_NK4A136_group was shown to reduce intestinal permeability and protect the intestinal epithelial barrier in mice [71]. We also observed that the abundance of Proteobacteria and *Prevotella*_9 increased, while the abundance of *Verrucomicrobia* and *Lachnospiraceae*_NK4A136_group decreased in UC rats. Moreover, YFBP treatment could reverse the above changes to some extent. Pharmacological studies found that caffeic acid in PJ could increase the abundance of intestinal beneficial bacteria *Akkermansia* and *Dubosiella* in UC mice induced by DSS [60]. Quercetin in PJ could regulate intestinal microbiota [72]. Kaempferol and luteolin in PJ could increase the abundance and diversity of intestinal microbiota in UC rats and reduce the abundance of pathogenic bacteria Proteobacteria, thereby remodeling intestinal microbiota structure [50, 73].

## 5. Conclusion

In conclusion, our study revealed that YFBP had a good therapeutic effect on UC, and its mechanism might be related to inhibiting the TLR4/NF-*κ*B/NLRP3 inflammasome signaling pathway and reducing the levels of inflammatory factors to repair intestinal epithelial barrier and modulating intestinal microbiota. These findings provide a new avenue for understanding the intestinal protective effects and potential mechanisms of YFBP on UC.

## Figures and Tables

**Table 1:** Details of the YFBP formula.

Species	Family	Vernacular name	Part used	TCM	Amount (g)	Batch code	Archive number
Coix lacryma-jobi var. ma-yuen (Rom. Caill.) Stapf	Poaceae Barnhart	Yi Yi Ren	Fruit	1	30	2104230022	21111508
*Aconitum carmichaelii* Debeaux	Ranunculaceae Juss	Fu Zi	Root	2	6	2107290572	21111509
*Patrinia villosa* (Thunb.) Juss	Valerianaceae	Bai Jiang Cao	Whole herb	3	15	210601	21111510

## Data Availability

All data included in this study are available upon request by contact with the corresponding author.

## Abbreviations

YFBP: Yiyi Fuzi Baijiang powder
UC: Ulcerative colitis
DSS: Dextran sulfate sodium
DAI: Disease activity index
DEGs: Differentially expressed genes
GO: Gene Ontology
KEGG: Kyoto Encyclopedia of Genes and Genomes
PPI: Protein-protein interaction network
TLRs: Toll-like receptors
NF-*κ*B: Nuclear factor-kappa B
TJ: Tight junction.

## Supplementary Materials

Supplementary Materials Supplementary Table 1: information on 40 drug ingredients in network pharmacology. Supplementary Table 2: molecular docking binding energy. Supplementary Table 3: body weight of rats in each group. Supplementary Table 4: the disease activity index (DAI) score in each group. Supplementary Table 5: colon length of rats in each group. Supplementary Table 6: the chemical constituents of YFBP-containing serum identified by UPLC-Q-Orbitrap HRMS. Supplementary Table 7: the protein expressions of TLR4/NF-*κ*B p65/NLRP3 inflammasome signaling pathway and tight junctions. Supplementary Table 8: the fluorescence intensity of NLRP3, GSDMD-N, and ZO-1. Supplementary Table 9: the levels of cytokines.
