# Supplementary material for: Yiyi Fuzi Baijiang Powder Alleviates Dextran Sulfate Sodium-Induced Ulcerative Colitis in Rats via Inhibiting the TLR4/NF-κB/NLRP3 Inflammasome Signaling Pathway to Repair the Intestinal Epithelial Barrier, and Modulating Intestinal Microbiota
Source: Oxid Med Cell Longev. 2023 Jan 14;2023:3071610. doi: 10.1155/2023/3071610 (PMC9867587; doi:10.1155/2023/3071610)
Supplement: Supplementary Materials — Supplementary Table 1: information on 40 drug ingredients in network pharmacology. Supplementary Table 2: molecular docking binding energy. Supplementary Table 3: body weight of rats in each group. Supplementary Table 4: the disease activity index (DAI) score in each group. Supplementary Table 5: colon length of rats in each group. Supplementary Table 6: the chemical constituents of YFBP-containing serum identified by UPLC-Q-Orbitrap HRMS. Supplementary Table 7: the protein expressions of TLR4/NF-κB p65/NLRP3 inflammasome signaling pathway and tight junctions. Supplementary Table 8: the fluorescence intensity of NLRP3, GSDMD-N, and ZO-1. Supplementary Table 9: the levels of cytokines. [file 3071610.f1.docx]

Table 1 Information on 40 drug ingredients in network pharmacology

| Herbs | MOL ID | Molecule name |
| --- | --- | --- |
| CS | MOL001323 | Sitosterol alpha1 |
| CS | MOL001494 | Mandenol |
| CS | MOL002372 | (6Z,10E,14E,18E)-2,6,10,15,19,23-hexamethyltetracosa-2,6,10,14,18,22-hexaene |
| CS | MOL002882 | [(2R)-2,3-dihydroxypropyl] (Z)-octadec-9-enoate |
| CS | MOL008118 | Coixenolide |
| CS | MOL008121 | 2-Monoolein |
| CS | MOL000953 | CLR |
| AD | MOL002211 | 11,14-eicosadienoic acid |
| AD | MOL002388 | Delphin_qt |
| AD | MOL002392 | Deltoin |
| AD | MOL002393 | Demethyldelavaine A |
| AD | MOL002394 | Demethyldelavaine B |
| AD | MOL002395 | Deoxyandrographolide |
| AD | MOL002397 | karakoline |
| AD | MOL002398 | Karanjin |
| AD | MOL002401 | Neokadsuranic acid B |
| AD | MOL002406 | 2,7-Dideacetyl-2,7-dibenzoyl-taxayunnanine F |
| AD | MOL002410 | benzoylnapelline |
| AD | MOL002415 | 6-Demethyldesoline |
| AD | MOL002416 | deoxyaconitine |
| AD | MOL002419 | (R)-Norcoclaurine |
| AD | MOL002421 | ignavine |
| AD | MOL002422 | isotalatizidine |
| AD | MOL002423 | jesaconitine |
| AD | MOL002433 | (3R,8S,9R,10R,13R,14S,17R)-3-hydroxy-4,4,9,13,14-pentamethyl-17-[(E,2R)-6-methyl-7-[(2R,3R,4S,5S,6R)-3,4,5-trihydroxy-6-[[(2R,3R,4S,5S,6R)-3,4,5-trihydroxy-6-(hydroxymethyl)oxan-2-yl]oxymethyl]oxan-2-yl]oxyhept-5-en-2-yl]-1,2,3,7,8,10,12,15,16,17-decahydr |
| AD | MOL002434 | Carnosifloside I_qt |
| PJ | MOL000538 | hypaconitine |
| PJ | MOL001676 | Vilmorrianine C |
| PJ | MOL001677 | asperglaucide |
| PJ | MOL001678 | bolusanthol B |
| PJ | MOL001790 | Linarin |
| PJ | MOL001689 | acacetin |
| PJ | MOL002322 | isovitexin |
| PJ | MOL001697 | Sinoacutine |
| PJ | MOL000358 | beta-sitosterol |
| PJ | MOL000422 | kaempferol |
| PJ | MOL000006 | luteolin |
| PJ | MOL000098 | quercetin |
| CA, AD and PJ | MOL000359 | sitosterol |
| CA and PJ | MOL000449 | Stigmasterol |

Table 2 Molecular docking binding energy

| Protein | MOLID | Ingredients | Sources | binding sites | Binding energy(kcal/mol) |
| --- | --- | --- | --- | --- | --- |
| IL1β | MOL002410 | Benzoylnapelline | AD | LEU-134 | -6.8 |
| IL1β | MOL000422 | Kaempferol | PJ | ARG-11  GLN-15 | -6.3 |
| JUN | MOL002410 | Benzoylnapelline | AD | SER-45  LYS-49  LYS-122  ASN-175 | -7.4 |
| MMP9 | MOL000098 | Quercetin | PJ | ALA-189  LEU-188  LEU-418 | -9.0 |
| MMP9 | MOL002419 | (R)-Norcoclaurine | AD | GLY-186  MET-422 | -8.6 |
| HIF1α | MOL000098 | Quercetin | PJ | ARG-312  GLY-349 | -8.3 |
| CCL2 | MOL000422 | Kaempferol | PJ | ARG-48  GLN-28 | -6.9 |
| TLR4 | MOL002397 | Karakoline | AD | GLY-70  SER-73  LYS-47 | -6.7 |

Table 3 Body weight of rats in each group （n=8）

| Days | Normal | DSS | DSS+YFBP |
| --- | --- | --- | --- |
| 1 | 223.68±6.530 | 224.36±10.864 | 227.69±5.444 |
| 2 | 231.70±8.938 | 230.03±8.521 | 231.54±3.639 |
| 3 | 248.34±12.320^*^ | 237.01±9.146 | 237.33±4.792 |
| 4 | 261.49±13.652^**^ | 243.78±8.880 | 243.80±5.039 |
| 5 | 268.88±14.649^**^ | 250.29±8.639 | 249.75±4.857 |
| 6 | 280.21±13.774^**^ | 256.71±9.124 | 255.89±4.862 |
| 7 | 294.06±12.810^**^ | 254.93±8.733 | 253.91±6.773 |
| 8 | 301.63±13.019^**^ | 250.06±8.619 | 249.18±6.987 |
| 9 | 309.73±13.782^**^ | 245.44±8.292 | 244.15±6.727 |
| 10 | 320.61±17.594^**^ | 240.09±8.467 | 238.76±6.401 |
| 11 | 331.73±18.467^**^ | 232.91±9.354 | 232.35±5.599 |
| 12 | 339.91±18.522^**^ | 236.23±9.679 | 238.14±6.245 |
| 13 | 350.39±17.718^**^ | 242.60±9.636 | 248.66±8.236 |
| 14 | 360.23±19.808^**^ | 249.11±10.615 | 259.96±8.197^#^ |
| 15 | 370.49±21.286^**^ | 255.75±10.133 | 272.58±7.929^##^ |
| 16 | 382.34±19.382^**^ | 262.94±10.150 | 285.60±7.374^##^ |
| 17 | 392.86±18.113^**^ | 270.35±9.089 | 298.88±6.653^##^ |

^*^*p* < 0.05, ^**^*p* < 0.01, Normal vs. DSS group; ^#^*p* < 0.05, ^##^*p* < 0.01, DSS+YFBP vs. DSS group.

Table 4 The disease activity index (DAI) score in each group (n=8)

| Days | Normal | DSS | DSS+YFBP |
| --- | --- | --- | --- |
| 1 | 0 | 0 | 0 |
| 2 | 0 | 0.25±0.154 | 0.21±0.248 |
| 3 | 0 | 0.41±0.154 | 0.38±0.118 |
| 4 | 0 | 0.54±0.306 | 0.46±0.248 |
| 5 | 0 | 0.67±0.357 | 0.63±0.278 |
| 6 | 0 | 0.92±0.233 | 0.88±0.246 |
| 7 | 0 | 1.04±0.215 | 1.04±0.277 |
| 8 | 0 | 1.33±0.473 | 1.29±0.214 |
| 9 | 0 | 1.50±0.357 | 1.46±0.306 |
| 10 | 0 | 1.79±0.354 | 1.79±0.354 |
| 11 | 0 | 2.08±0.295 | 2.08±0.389 |
| 12 | 0 | 1.67±0.253 | 1.50±0.357 |
| 13 | 0 | 1.21±0.171 | 0.96±0.211^#^ |
| 14 | 0 | 0.83±0.254 | 0.54±0.250^#^ |
| 15 | 0 | 0.67±0.357 | 0.29±0.214^#^ |
| 16 | 0 | 0.58±0.238 | 0.21±0.171^##^ |
| 17 | 0 | 0.50±0.181 | 0.12±0.171^##^ |

^#^*p* < 0.05, ^##^*p* < 0.01, DSS+YFBP vs. DSS group.

Table 5 Colon length of rats in each group (n=8)

| Group | Length |
| --- | --- |
| Normal | 19.36±1.686^**^ |
| DSS | 14.90±1.161 |
| DSS+YFBP | 17.22±0.577^##^ |

^**^*p* < 0.01, Normal vs. DSS group; ^##^*p* < 0.01, DSS+YFBP vs. DSS group.

Table 6 The chemical constituents of YFBP-containing serum identified by UPLC-Q-Orbitrap HRMS

| Num-ber | Name | Formula | Molecular Weight | RT (min) |
| --- | --- | --- | --- | --- |
| 1 | Methyl 1-(hexopyranosyloxy)-5-hydroxy-7-(hydroxymethyl)-1,4a,5,7a-tetrahydrocyclopenta[c]pyran-4-carboxylate | C17 H24 O11 | 404.13148 | 10.678 |
| 2 | 6-Ketoprostaglandin F1α | C20 H34 O6 | 352.22213 | 16.622 |
| 3 | Caffeic acid | C9 H8 O4 | 180.04205 | 18.792 |
| 4 | Pinolenic acid | C18 H30 O2 | 278.22437 | 21.79 |
| 5 | γ-Linolenic acid ethyl ester | C20 H34 O2 | 312.26578 | 22.195 |
| 6 | Palmitic acid | C16 H32 O2 | 256.23991 | 22.931 |
| 7 | trans-Petroselinic acid | C18 H34 O2 | 282.25574 | 23.276 |

Table 7 The protein expressions of TLR4/NF-κB p65/NLRP3 inflammasome signaling pathway and tight junctions (n=3)

|  | Normal | DSS | DSS+YFBP |
| --- | --- | --- | --- |
| TLR4 | 0.52±0.026 | 1.13±0.127^**^ | 0.90±0.117^#^ |
| p-NF-κB p65 | 0.63±0.020 | 1.08±0.096^**^ | 0.87±0.093^#^ |
| NLRP3 | 0.31±0.078 | 0.75±0.082^**^ | 0.57±0.097^#^ |
| ASC | 0.38±0.069 | 1.00±0.161^**^ | 0.71±0.109^#^ |
| cleaved caspase-1 | 0.35±0.034 | 0.81±0.036^**^ | 0.49±0.079^##^ |
| GSDMD-N | 0.27±0.033 | 0.97±0.078^**^ | 0.55±0.081^##^ |
| ZO-1 | 1.08±0.115 | 0.65±0.035^**^ | 0.82±0.084^#^ |
| Occludin | 1.15±0.088 | 0.83±0.025^**^ | 1.10±0.147^#^ |
| Claudin 1 | 1.12±0.110 | 0.61±0.093^**^ | 0.91±0.059^##^ |

^**^*p* < 0.01 vs. Normal group; ^#^*p* < 0.05, ^##^*p* < 0.01 vs. DSS group.

Table 8 The fluorescence intensity of NLRP3, GSDMD-N, and ZO-1 (n=3)

|  | Normal | DSS | DSS+YFBP |
| --- | --- | --- | --- |
| NLRP3 | 29.73±4.662 | 104.17±5.006^**^ | 65.80±13.905^##^ |
| GSDMD-N | 34.75±12.706 | 110.12±9.150^**^ | 53.31±10.265^##^ |
| ZO-1 | 134.21±9.807 | 48.33±2.767^**^ | 83.91±9.012^##^ |

^**^*p* < 0.01 vs. Normal group; ^##^*p* < 0.01 vs. DSS group.

Table 9 The levels of cytokines (pg/g, n=3)

| Group | IL-Iβ | TNF-α |
| --- | --- | --- |
| Normal | 1351.11±245.646 | 850.90±97.817 |
| DSS | 3266.67±280.423^**^ | 2151.79±182.037^**^ |
| DSS+YFBP | 2079.44±216.244^##^ | 1421.03±137.441^##^ |

^**^*p* < 0.01 vs. Normal group; ^##^*p* < 0.01 vs. DSS group.
